# Addressing the burden of violence on global mental health: contributions of Narrative Exposure Therapy across different health systems

**DOI:** 10.1590/0102-311XEN199124

**Published:** 2025-10-03

**Authors:** Fernanda Serpeloni, Anke Köbach, Amani Chibashimba, Anselm Crombach, Itsuko Domen, Iván Arango, Joviana Quintes Avanci, Katy Robjant, Liliana Abreu, Maggie Schauer, Nathalie Görtz, Susanne Axelsson, Vanessa Nolasco Ferreira, Simone Gonçalves de Assis

**Affiliations:** 1 Fundação Oswaldo Cruz, Rio de Janeiro, Brasil.; 2 vivo international e.V., Konstanz, Germany.; 3 University of Konstanz, Konstanz, Germany.; 4 University of Saarland, Saarbrücken, Germany.; 5 Kansai University of International Studies, Kobe, Japan.; 6 Instituto Nacional de Psiquiatría Ramón de la Fuente Muñiz, Ciudad de México, México.; 7 Kristiania University of Applied Sciences, Oslo, Norway.

**Keywords:** Post-Traumatic Stress Disorders, Mental Health, Violence, Trauma, Global Health, Trastornos de Estrés Postraumático, Salud Mental, Violencia, Trauma, Salud Global

## Abstract

Post-traumatic stress disorder represents a substantial global mental health burden, particularly in the face of cumulative violence, forced migration, and structural inequities. Narrative Exposure Therapy (NET) configures a brief trauma-focused intervention that supports the reconstruction of autobiographical memory by the chronological narration of life events. By integrating fragmented traumatic experiences into a coherent narrative, NET facilitates emotional processing and restores continuity to disrupted life stories. This regional case series examines the integration of NET into the mental health systems in Brazil, the Democratic Republic of Congo, Germany, Switzerland, Japan, Mexico, the United Kingdom, and Scandinavia. Drawing on diverse implementation experiences, the study identifies both enabling conditions and persistent challenges. Results highlight that NET is feasible and adaptable across different sociocultural and resource settings, especially when supported by sustained supervision, task-shifting strategies, and intersectoral collaboration. Embedding NET into existing service structures expanded access to evidence-based trauma care for populations often excluded from specialized treatment. These findings underscore the critical role of trauma-informed public policies in responding to the mental health consequences of violence on a global scale.

## Abstract (es)

El trastorno de estrés postraumático (TEPT) representa una carga sustancial para la salud mental mundial, particularmente en contextos moldeados por la violencia acumulativa, la migración forzada y las desigualdades estructurales. La Terapia de Exposición Narrativa (NET, por su sigla en inglés) es una intervención breve centrada en el trauma que apoya la reconstrucción de la memoria autobiográfica a través de la narración cronológica de eventos de la vida. Al permitir la integración de experiencias traumáticas fragmentadas en una narrativa coherente, NET facilita el procesamiento emocional y restaura la continuidad de las historias de vida interrumpidas. Esta serie de casos regionales examina la integración de las NET en los sistemas de salud mental de Brasil, la República Democrática del Congo, Alemania, Suiza, Japón, México, el Reino Unido y los países escandinavos. Sobre la base de diversas experiencias de implementación, el estudio identifica tanto las condiciones propicias como los desafíos persistentes. Los resultados ponen de relieve que la NET es factible y adaptable en diversos entornos socioculturales y con distintos recursos, especialmente cuando está respaldada por una supervisión sostenida, estrategias de cambio de tareas y colaboración intersectorial. La incorporación de NET en las estructuras de servicios existentes amplió el acceso a la atención del trauma basada en la evidencia para las poblaciones a menudo excluidas de los tratamientos especializados. Estos hallazgos subrayan el papel fundamental de las políticas públicas informadas sobre el trauma para responder a las consecuencias de la violencia en la salud mental a escala mundial.

## Introduction

Violence has a significant global health impact, contributing to the development of trauma-related disorders, particularly post-traumatic stress disorder (PTSD). Globally, about 70% of people experience at least one potentially traumatic event during their lifetime, with approximately 5.6% subsequently developing PTSD ^1^. The global burden of violence-related mental health problems poses a significant challenge, particularly among populations affected by armed conflict, forced displacement, or chronic community violence − contexts where trauma exposure is not a singular occurrence but a cumulative life condition ^2^.

PTSD can have profound and enduring effects on individuals, significantly impairing their overall functioning and well-being. Common symptoms include recurrent intrusive memories, emotional numbing, hypervigilance, and physiological arousal, often resulting in functional impairment and social withdrawal ^3^. Comorbidities such as depression and anxiety are prevalent among individuals with PTSD, further exacerbating the overall health burden ^4^. These symptoms can severely impact a person’s ability to function in professional, domestic, and social settings. PTSD is a leading contributor to disability-adjusted life years (DALYs) worldwide ^5^. Research has also linked traumatic events to a range of physical health issues, including chronic pain, cardiovascular diseases, and immune system dysfunction ^6^. In many settings, especially in low- and middle-income countries, trauma survivors remain untreated due to stigma, shortage of trained providers, and structural barriers to accessing care. The magnitude of this treatment gap constitutes an urgent global health priority.

While evidence-based psychotherapeutic interventions such as trauma-focused cognitive behavioral therapy ^7^, Narrative Exposure Therapy (NET) ^8^ ^,^ ^9^, and eye movement desensitization and reprocessing (EMDR) ^10^ are available, NET stands out for its unique approach to trauma treatment, combining storytelling and memory processing. This makes it particularly effective for individuals who have experienced multiple or complex traumatic events such as childhood abuse or human rights violations. NET is a short-term, evidence-based, and culturally sensitive treatment ^8^ ^,^ ^9^. This approach was designed to be implemented even by individuals without specialized mental health training, making it well-suited for humanitarian and low-resource contexts. NET is manualized and typically involves 6-12 sessions. Adaptations of NET have been developed for specific populations, including children and adolescents (KIDNET) ^11^, traumatized offenders (FORNET) ^12^, and entire communities (NETfacts) ^13^. Clinical trials have shown sustained improvements in PTSD and depressive symptoms, even in environments marked by persistent insecurity ^14^.

NET is based on the understanding that trauma survivors often experience multiple adverse life events (“building blocks”), resulting in fragmented and disorganized trauma memories. NET guides clients in creating a chronological life narrative that highlights traumatic experiences while also incorporating significant positive events. With the therapist acting as an active listener, clients engage in meaning-making and are able to contextualize their sensory, cognitive, emotional, and physiological/bodily memories, addressing the pathological memory structures associated with their symptoms. NET encourages individuals to reflect on their life story, fostering a sense of personal identity and agency ^8^ ^,^ ^9^.

This study aims to examine how NET has been integrated into diverse health systems globally, based on the practical experiences of therapists and program leaders. Rather than presenting isolated case studies, we adopt a comparative approach that enables the identification of both convergent patterns and context-specific adaptations. This methodological approach aligns with recommended practices in Global Mental Health ^15^ ^,^ ^16^, enabling the identification of common patterns and contextual differences among countries with diverse cultures. Furthermore, it offers critical insight into how trauma care can be scaled equitably across systems with differing levels of institutional support, resource availability, and social recognition of trauma as a public health concern.

## Methods

This regional report is based on the authors’ practice in various countries ^17^. Data were drawn from health systems of Brazil, the Democratic Republic of Congo, Germany, Switzerland, Japan, Mexico, the United Kingdom, and Scandinavia, which were selected using a snowball sampling method (Box 1). In each country, a NET project leader or therapist responded to a set of open-ended questions addressing: a brief characterization of the country’s health system; the background of participating therapists; characteristics of clients regarding their traumatic experiences; and challenges faced during NET implementation. All representatives who responded to the script are co-authors of this manuscript and contributed to the critical analysis. The goal was to gain theoretical insight into situations arising from their professional experience; therefore, approval from a research ethics committee was not required. No identifiable data or participant information is disclosed. Respondents included both health professionals formally embedded in national systems and community-based practitioners. This diversity reflects NET’s flexible implementation potential, supporting task-shifting and integration into established services, as recognized in global health frameworks ^18^. The term task shifting is used by the World Health Organization (WHO) ^18^ to describe a health workforce reorganization strategy that involves redistributing tasks among different professional categories, with the aim of expanding service access and coverage in contexts of human resource shortages. For its effectiveness, task shifting requires investments in training, supervision, and institutional support, as well as public policies that ensure the quality and comprehensiveness of care. Consistent with principles of global mental health research ^16^ ^,^ ^19^, we chose not to present isolated case studies but to employ a comparative lens that reveals both convergent and divergent patterns. This methodological decision aligns with recommendations for synthesis over fragmentation in cross-national contexts in which scalable mental health interventions are needed ^18^.

**Box 1:** Summary of Narrative Exposure Therapy (NET) implementation across countries.

COUNTRY	IMPLEMENTATION MODEL	NET THERAPISTS	RELATION TO HEALTH SYSTEM	CLIENTS	KEY IMPLEMENTATION INSIGHTS
Brazil	Formal in public primary care and judicial services	Psychologists, family physicians, psychiatrists, social workers	Public health professionals (SUS)	Survivors of sexual abuse, intimate partner violence, urban violence	Need for specialized trauma services within health systems. Coordination between public health and the justice system as an entry point for a continuum of care for survivors of violence
Democratic Republic of Congo	Community-based via NGOs	Community counselors	Outside formal health system	Survivors of gender-based violence, former combatants	Community model enabled scalability
Mexico	Formal in national psychiatric institutions	Psychologists, psychiatrists	National public institution	Survivors of sexual abuse, interpersonal violence	Integration without protocol adaptation
Germany	Formal in public/private outpatient services	Psychologists, psychiatrists, social workers, others	Public and private practitioners	Refugees, asylum seekers, domestic violence survivors	Insurance supports broader implementation
Switzerland	Specialized forensic clinical integration	Psychologists, forensic clinicians	Specialized public forensic practitioners	Forensic clients with severe mental illness	Forensic adaptation restores resilience
United Kingdom	Hybrid NHS and NGOs	Psychologists, psychiatrists, nurses, social workers, therapists	Public NHS and NGO staff	Survivors of abuse, war, trafficking	Training expanded IAPT staff reach
Japan	Private and institutional clinical practice	Psychologists, psychiatrists	Private sector, academic	Survivors of abuse, accidents, disasters	Preliminary evidence of symptom reduction
Norway	Specialist services and community-level public initiatives	Psychologists, psychiatric nurses, social workers	National health services	Refugees, individuals with substance use disorders	Culturally matched delivery and integration with substance recovery services
Sweden	Local municipal partnerships and community-based organizations	Psychologists, NET-trained therapists	Public sector and local initiatives	Survivors of war, migration, interpersonal and organized violence	Community-based care with public engagement (e.g., theater, stigma reduction)

IAPT: Improving Access to Psychological Therapies; NGO: non-governmental organizations; NHS: National Health System; SUS: Brazilian Unified National Health System.

Source: prepared by the authors.

Note: this box synthesizes key characteristics of NET implementation in diverse contexts, focusing on integration models, therapists, and client profiles to support comparative analysis across settings.

## Results

### Experiences of implementing NET in various countries

#### Brazil

Brazil’s healthcare system comprises both public and private sectors. The Brazilian Unified National Health System (SUS, acronym in Portuguese) offers universal, comprehensive, and free healthcare services, emphasizing equity and decentralization. Primary care settings are a critical entry point for individuals experiencing PTSD, who often present with symptoms such as sleep problems, panic attacks, hypertension, and depression ^20^. However, PTSD is frequently undiagnosed in primary care, contributing to suboptimal treatment and high costs ^21^. This underscores the need to better integrate trauma-focused therapies such as NET into the public health system.

Since 2018, a multidisciplinary team has advanced the implementation of NET in Brazil with feasibility studies and clinical trials conducted within public services ^22^ ^,^ ^23^ ^,^ ^24^. Family physicians, nurses, psychiatrists, and psychologists were trained to deliver NET in two-week sessions, followed by regular supervision. Clients had experienced severe and often cumulative forms of trauma, such as urban violence, childhood abuse, and sexual and domestic violence, with many continuing to face threats during or after treatment. The results showed a clinical reduction in PTSD symptoms across all settings, even amid continuous violence ^22^. Furthermore, consistent with the principles of NETfacts, a music video was developed based on client’s composite narrative to reduce stigma and shift attitudes toward domestic violence (https://www.youtube.com/watch?v=iaUm7tcAoEA).

FORNET has been implemented in Judicial Centers for Conflict Resolution ^25^ and penitentiaries. NET was well accepted by professionals during its integration into standard care; however, systemic constraints persist. The length of NET sessions was difficult to accommodate within high-turnover primary care settings. The integration of FORNET into penitentiaries is ongoing, showing promising results. Brazil’s case illustrates both the potential and limitations of embedding NET within a formally structured but overstretched health system. These lessons underscore the need for policy-level interventions to support task-shifting, trauma recognition, and financing mechanisms for specialized services. Such elements are essential to enable sustainable, scalable trauma care in contexts marked by structural violence and social inequality.

#### Democratic Republic of Congo

The Democratic Republic of Congo has experienced decades of armed conflict and mass displacement, leading to a significant burden of trauma-related disorders among its population. The Congolese health system, governed by the Ministry of Health, comprises provincial health divisions and local health zones, which serve as the primary implementation units. Initial attempts to integrate NET into the primary care system − by training nurses in conflict-affected provinces − were thwarted by chronic health workforce shortages and operational overload. Consequently, the model shifted toward a community-based implementation using local non-governmental organizations (NGOs) and community-based organizations (CBOs). This shift illustrates an adaptive approach to the implementation of NET that prioritized local ownership, trust, and sustainability. Community counselors, many of whom were survivors themselves, were trained using a train-the-trainer model to ensure continuity and scalability of services. Prior to NET training, CBOs received basic training in supportive counseling, gender-based violence, and referral protocols, enabling community-level counselors to deliver treatment and document cases within the reporting framework of the health zone.

The project series primarily targeted individuals aged 16 years and older. Most participants had experienced gender-based violence and other traumatic events during prolonged armed conflicts, and many had also perpetrated acts of violence. In addition to individual NET treatment, the efficacy of adapting NET to communities with NETfacts was investigated ^13^ ^,^ ^26^. The use of NETfacts at the community level, along with tiered supervision (counselors, master counselors, and supervisor counselors), is a robust innovation in task-shifting and decentralized trauma care. This structure not only facilitated psychosocial recovery but also promoted collective meaning-making and reconciliation. The trainings ranged from three to seven weeks, including a three-week NET/FORNET training with a two-week refresher, a two-week master counselor training plus one week of additional training, and a three-week NETfacts training. This community-based approach strengthened the health system by reinforcing mental health care and awareness at the grassroots level. However, recurrent political instability, violence, and the displacement of trained personnel disrupted service continuity. Despite these barriers, the DRC experience demonstrates how NET can be mobilized with the aid of community networks in sensitive settings, offering lessons relevant for or resource-limited countries.

#### Mexico

The National Institute of Psychiatry “Ramón de la Fuente Muñiz” in Mexico, a national psychiatric reference center within the National Network of Health Institutes, offers NET to clients from across the country, primarily from Mexico City and surrounding states. As a public institution, the Institute provides treatment free of charge or at a subsidized fee, depending on clients’ socioeconomic status. The integration of NET has occurred without the need for protocol adaptation, demonstrating its compatibility with structured mental health services. NET therapists come from diverse theoretical orientations, including cognitive behavioral therapy, family systems, acceptance and commitment therapy, and transference-focused approaches. Clients present with a range of psychiatric comorbidities in addition to PTSD and complex trauma, such as major depressive disorder, anxiety disorders, substance use disorders, personality disorders, and perinatal psychiatric disorders. Many have experienced various forms of trauma, including sexual abuse, interpersonal and partner-related violence, obstetric violence, and urban violence. The NET training program at the Institute typically lasts four to five days and was initially provided by the NET Institute at the University of Konstanz (Germany). 

Institutional conditions such as training infrastructure, clinical supervision, and interdisciplinary collaboration have contributed to a consistent, high-fidelity NET delivery. Clients have shown improvements across emotional, cognitive, and somatic domains. Importantly, the therapeutic process incorporates a gender-sensitive approach, reflecting growing attention to intersectional dimensions of trauma ^27^. This model highlights the clinical viability and therapeutic efficacy of NET in specialized care, while also underscoring a broader challenge in global mental health: ensuring equity and scalability beyond specialized urban centers.

#### Germany and Switzerland

Germany has a well-established healthcare system, underpinned by mandatory health insurance since 2009, which ensures nearly universal coverage for its population of approximately 83 million. The two-tier system is primarily divided into statutory health insurance (SHI) and private health insurance (PHI). About 86% of the population is enrolled in SHI, which is financed by income-based contributions shared between employers and employees ^28^, while about 10%-11% is covered by PHI. This system provides comprehensive coverage, including inpatient, outpatient, mental health, and prescription drug services ^29^. Despite its strengths, the German healthcare system faces challenges such as fragmentation between outpatient and inpatient care, which can delay integrated treatment approaches ^28^. The Swiss healthcare system is similar to that in Germany, but with greater freedom of choice regarding specific services and direct access to all levels of care. In both countries, General practitioners are crucial in providing support and counseling for psychosomatic illnesses and in caring for individuals with mental health issues ^30^. However, they typically do not offer regular psychotherapy or other specialized psychological interventions. Access to these specialized services is often limited, with many clients facing difficulties in obtaining timely and appropriate care. 

NET is one of the trauma-focused therapies accepted in Germany and is particularly effective in treating trauma spectrum disorders. It has gained traction due to its structured, narrative-driven approach ^31^ to addressing multiple traumas. Both insurance companies and public health services cover NET. Given Germany’s significant refugee population, NET has been increasingly used to support trauma-affected refugees. Many practitioners in refugee centers and specialized clinics use NET because of its adaptability to these contexts. Over the past decades, however, NET has become an increasingly important treatment option to the local population ^32^ ^,^ ^33^ ^,^ ^34^ ^,^ ^35^. German universities and research institutions, such as the University of Konstanz, have contributed to NET research. This evidence supports its effectiveness and has contributed to its broader acceptance within insurance-covered treatments and by healthcare providers.

The integration of NET into the German and Swiss healthcare systems involved several key steps. Initially, stakeholders − including mental health professionals, NGOs, and health policymakers − were engaged to assess the need for trauma-focused interventions. Training programs were established to ensure that therapists were adequately prepared to deliver NET effectively. Collaboration with various healthcare institutions facilitated the incorporation of NET into treatment pathways for individuals experiencing trauma, particularly refugees and violence survivors. Therapists delivering NET, KIDNET, and FORNET come from diverse backgrounds, primarily clinical psychologists and psychiatrists, but also social workers and others. NET is now offered in a range of settings, including private practice, outpatient clinics, inpatient wards, and specialized institutions. The duration of NET training programs typically spans from two days to several weeks, including both theoretical and practical components to ensure therapists are adequately prepared to implement the intervention. The profile of individuals receiving NET includes refugees and asylum seekers, as well as survivors of sexual abuse, domestic violence, natural disasters, accidents, and other traumatic experiences. 

All clients have experienced significant trauma or violence, including childhood abuse, domestic violence, mobbing, peer violence, war-related experiences, displacement, and interpersonal violence ^36^ ^,^ ^37^ ^,^ ^38^. A recent multicenter randomized controlled trial ^39^ assessed the feasibility of integrating NET into primary care, focusing on the role of general practioners teams in its practical implementation. The findings were encouraging, with good acceptance of NET when integrated into standard care. A unique project at the Münsterlingen Forensic Clinic in Switzerland uses NET with traumatized clients with severe mental illness (e.g. schizophrenia, bipolar affective disorder) who have been convicted or acquitted of interpersonal crimes. The project recognizes that cumulative exposure to violence can impair phycological adjustment and contribute to the development of mental health symptoms. 

In Germany, trauma-informed and trauma-memory-focused mental health care using NET ^40^ is progressively being expanded with the inclusion of professionally trained lay personnel as part of the narrative trauma work (NAT) intervention ^41^. These individuals collaborate with traditional psychotherapists in a stepped-care approach to befriend, identify, assess, and support potential individuals in need, engaging in biographical work. The experience in Germany and Switzerland reveals how NET can be integrated into highly structured systems, facilitating its incorporation into both specialized services and private practice. While initially developed for refugees and conflict-affected populations, NET is now routinely used with individuals from the general population suffering from trauma. In Germany, the inclusion of NET within both public and private insurance schemes has facilitated its broader institutionalization. Meanwhile, Switzerland has demonstrated its effective application in specialized settings such as forensic psychiatric care. These cases highlight how policy alignment, insurance integration, and training infrastructure are essential for scaling evidence-based trauma therapy. 

#### United Kingdom

The United Kingdom National Health Service (NHS) provides mental health care using a stepped care model accessible to all residents, including asylum seekers and refugees. Initial access is typically via primary care and the Improving Access to Psychological Therapies (IAPT) program. More severe cases are referred to secondary or tertiary care services. Specialist trauma services across the United Kingdom offer evidence-based treatments for PTSD and complex PTSD, with NET now routinely integrated into these services. Forensic services have been trained to offer FORNET, and child and adolescent mental health services have incorporated KIDNET into their repertoire. In addition to NHS services, several NGOs offer evidence-based trauma treatments, with those working with asylum seekers, refugees, and survivors of trafficking being specifically targeted for NET training. In the early years of NET implementation in the United Kingdom, tertiary specialist trauma services were the main focus for training. More recently, efforts have shifted towards training cohorts of IAPT healthcare staff as part of a more comprehensive training program. This initiative includes “top-up” training designed to ensure therapists feel confident working with asylum seekers and refugees, in collaboration with NGOs and an NHS Healthcare Trust. This has expanded the reach and availability of evidence-based trauma treatments at the primary care level, ensuring that asylum seekers and refugees are able to access these services. 

NET therapists in the United Kingdom come from diverse backgrounds, including clinical and counselling psychologists, forensic psychologists, psychiatrists, mental health nurses, art and music therapists, occupational therapists, social workers, psychotherapists, and counsellors, working across NHS and NGO services. Training usually ranges from two to five days, depending on the needs of the service and the practitioner’s experience, and is consistently followed by clinical supervision. Clients include British-born individuals who have experienced developmental and multiple trauma, British veterans, asylum seekers, refugees, and survivors of trafficking who have experienced developmental and multiple trauma in their country of origin, during migration, and within the United Kingdom. Children have received KIDNET and clients of forensic services have received FORNET, while United Kingdom veterans have been treated with FORNET or NET. The types of trauma and violence experienced by individuals treated with NET, FORNET, or KIDNET includes multiple forms of child abuse, gang violence, institutional abuse, war and conflict-related trauma, trafficking, torture, gender-based violence (GBV) − including forced marriage, female genital mutilation, sexual abuse, and rape. Treatment has also been provided to individuals who have perpetrated violence (whether as children or adults) including former child soldiers, gang members, and combatants, as well as those affected by intimate partner violence. The United Kingdom provides a unique example of a hybrid implementation strategy that bridges public sector delivery (via the NHS) with NGO-led initiatives. Originally targeted at asylum seekers, refugees, and survivors of trafficking, NET has since expanded into a range of services, including IAPT, forensic units, veteran support programs, and child and adolescent mental health services. Training pathways have been adapted to different service levels, with brief courses and supervision tailored to staff experience, thereby ensuring both quality and scalability. This model illustrates how structured health systems can flexibly incorporate trauma-specific interventions when combined with clear national guidance, strategic partnerships, and ongoing supervision. Successful implementation of NET and its adapted formats across various trauma services has contributed to its broader acceptance as an appropriate treatment for complex PTSD.

#### Japan

Japan operates under a universal health insurance system that ensures all citizens have access to medical care without financial barriers. This framework encompasses a broad spectrum of services; however, psychotherapy remains only partially covered. Within the existing reimbursement model for private practice physicians, psychotherapy is limited to a maximum of two sessions per month. This limitation hampers effective trauma treatment, which commonly requires weekly sessions to achieve optimal outcomes.

Currently, a research initiative is underway to explore NET implementation in the Japanese healthcare system. NET therapists include clinical psychologists, licensed psychologists, psychiatrists, psychiatric social workers, and school counselors − professionals already engaged in trauma care as part of their daily practice. Given their existing expertise in counseling, the NET training program spans three days and follows the international standard established by the NET Institute (https://www.net-institute.org/). After training, therapists implement NET under supervision for a designated period. These training efforts aim to address the significant shortage of trauma-informed care in Japan and build capacity within the mental health workforce. The goal is to support individuals affected by diverse traumatic experiences, such as child abuse, domestic violence, crime, sexual assault, traffic accidents, and natural disasters. 

Preliminary data from the ongoing research initiative in Japan suggest that the integration of NET has yielded promising results. Clients who have undergone NET report a significant reduction in PTSD symptoms, with many experiencing clinically meaningful improvement. These early outcomes are particularly notable among individuals affected by sexual violence, child abuse, and natural disasters. However, structural barriers remain: NET’s session duration and frequency are often incompatible with reimbursement policies, making sustained delivery difficult outside research or academic institutions. The Japanese experience reflects a broader disconnect between innovation in trauma care and the systemic readiness to accommodate intensive, evidence-based psychotherapies in national health systems. Policy reform, funding realignment, and increased recognition of trauma-related disorders are needed to translate promising pilot outcomes into widespread clinical practice.

#### Scandinavia

The Norwegian mental health system is publicly funded and structured to ensure universal healthcare. NET has been integrated into specialist services and public community-level initiatives ^42^. Its implementation involved national collaborations that trained culturally matched professionals to support Ukrainian refugees in their native language, enhancing accessibility and reducing pressure on specialist services. NET was also incorporated into structured inpatient services for individuals recovering from severe substance use, demonstrating its adaptability within multidisciplinary treatment models. Training and supervision are coordinated by national institutions, aligning with national mental health strategies. This approach underscores the role of trauma-informed care in addressing both forced displacement and comorbid substance use disorders. In Sweden, NET has been piloted via local municipal partnerships and community-based organizations. The initiative combined individual and group NET delivery with public education efforts to reduce stigma and raise awareness about trauma. A key part of the intervention was the community theater production *Barrikad*, based on the narratives of participants. The script was developed in close collaboration with service users, ensuring their voices and insights shaped the performance. Therapists trained in NET also provided training to other professionals, expanding service capacity. Participants included individuals exposed to war, migration, interpersonal violence, and organized crime. Reported outcomes included reduced PTSD symptoms, improved self-image, and greater peer support. Despite promising results, ensuring continuity of trauma-informed services remains a challenge. 

## Discussion

This regional study synthesizes the integration of NET across nine national health systems, highlighting both the feasibility and contextual challenges of implementing trauma-focused interventions. The findings underscore NET’s adaptability − ranging from centralized institutional models to decentralized community-based approaches − and highlight its capacity to reduce trauma-related symptoms in settings marked by violence, resource limitations, or systemic fragmentation.

Box 1 provides a comparative overview of implementation models, therapist profiles, and health system integration pathways. Countries such as Germany, the United Kingdom, and Mexico demonstrate how structured public systems can institutionalize NET in specialized services, whereas the Democratic Republic of Congo exemplify task-shifting and community-based dissemination. These variations reflect differing levels of systemic readiness, workforce capacity, and existing mental health governance structures. This reinforces WHO ^18^ recommendations for context-responsive models that balance clinical rigor with sociopolitical realities.

Despite clinical success, a recurring challenge across countries was the tension between model fidelity and system scalability. In Brazil and Japan, for instance, NET’s session length conflicted with the workflow of general health services and national reimbursement policies. This reflects a structural misalignment between evidence-based psychotherapy protocols and the operational logic of most public health systems.

To ensure equitable access, trauma-focused interventions must be supported by national policies, adequate financing, and clinical supervision systems that reach beyond urban tertiary centers. 

Mexico’s experience illustrates the clinical effectiveness of NET in specialized care and the urgent need to integrate trauma-focused approaches into the broader mental health system. Decentralized implementation offers a viable strategy to expand trauma-informed mental health services in vulnerable regions ^16^ ^,^ ^18^ ^,^ ^43^. Primary care, school-based services, and NGO platforms are critical entry points for scalable, decentralized delivery models. Global recommendations emphasize that the meaningful transformation of mental health systems requires task-shifting, policy advocacy, and financial mechanisms to support trauma-informed care beyond academic and urban contexts ^43^.

Cultural sensitivity and contextual adaptability have been central to the effective integration of NET across health systems. Language and cultural tailoring are among the most frequent modifications. Post-training supervision remains critical to ensure treatment fidelity and long-term sustainability. In the Democratic Republic of Congo, initial implementation combined intensive training with structured peer supervision and the development of a local network of trained practitioners. This strategy reinforced quality assurance, strengthened local capacity, and promoted ownership within the health system.

The Scandinavian experiences demonstrate that even structured systems may face challenges in institutionalizing trauma-focused care. In Norway, implementation occurred with coordinated efforts across specialist services and publicly funded community-level initiatives. This highlights how policy infrastructure, when combined with supervision systems and long-term planning, can facilitate the sustainable integration of NET. 

In Sweden, public awareness strategies, including participatory initiatives such as community theater, were integrated with clinical care to reduce stigma and foster engagement. While these approaches showed promising outcomes in symptom reduction and social reintegration, the lack of a centralized trauma strategy limited scalability. 

Together, the Scandinavian cases underscore that successful implementation, depends on intersectoral coordination, funding, and policy frameworks that explicitly recognize trauma as a public health priority. 

While traumatic stress is a growing global concern, the literature on trauma and PTSD remains disproportionately dominated by research conducted in high-income countries ^44^. This geographic bias limits the representativeness of the evidence base and fails to adequately capture the experiences and mental health needs of populations in low- and middle-income countries where the burden of violence, conflict, and other forms of adversity is often greatest. Addressing this imbalance is crucial for developing comprehensive, culturally-appropriate, and globally-relevant approaches for the prevention and treatment of trauma-related disorders.

From a global mental health perspective, NET is a culturally responsive intervention for trauma-related disorders, especially in low-resource and post-conflict settings. Its brief, structured format and capacity to address multiple traumatic events align with the needs of populations exposed to war, organized violence, and natural disasters. Its narrative-based approach resonates with universal storytelling practices, enhancing cultural relevance. Furthermore, NET’s integration of human rights documentation may carry broader socio-political implications in some contexts ^8^ ^,^ ^9^. 

As global mental health efforts strive to improve access to evidence-based treatments, NET’s adaptability and effectiveness make it a valuable tool. Rooted in robust research ^14^, NET should be accessible in all countries, including resource-poor contexts. Its training duration and intensity remain flexible, tailored to the therapist’s prior education and experience. By emphasizing task-shifting over strict regulation, NET is particularly suited for contexts of high demand, emergencies, or limited resources, facilitating timely dissemination.

A key limitation of this experience report is its restricted geographical scope, focusing on a limited number of countries. Meta-analyses indicate that NET clinical trials have been conducted across 30 countries ^14^, suggesting the potential for even broader implementation. Consequently, further research is warranted to enhance the understanding of NET implementation across diverse national contexts.

Methodologically, this study adopted a comparative approach to identify convergences and divergences across national experiences. This strategy aligns with recommendations from global mental health literature for integrative, rather than fragmented, implementation research ^15^ ^,^ ^18^. The use of a structured reporting framework, reflected in Box 1, facilitated cross-site analysis while preserving attention to each country’s sociopolitical and institutional context.

Overall, the evidence supports NET as a clinically effective and structurally flexible approach to trauma care. Its non-commercial design, adaptability to task-shifting, and alignment with global health equity goals make it a promising candidate for national scale-up efforts. However, successful implementation depends not only on therapeutic efficacy but also on systemic investment, workforce development, and context-specific innovation.
