# Supplementary material for: Addressing the burden of violence on global mental health: contributions of Narrative Exposure Therapy across different health systems
Source: Cad Saude Publica. 2025 Oct 3;41(9):e00199124. doi: 10.1590/0102-311XEN199124 (PMC12494385; doi:10.1590/0102-311XEN199124)
Supplement: Translation

# Translation: Os impactos da violência sobre a saúde mental global: contribuições da Terapia de Exposição Narrativa em diferentes sistemas de saúde

**DOI:** 10.1590/0102-311XPT199124

**Authors:** Fernanda Serpeloni, Anke Köbach, Amani Chibashimba, Anselm Crombach, Itsuko Domen, Iván Arango, Joviana Quintes Avanci, Katy Robjant, Liliana Abreu, Maggie Schauer, Nathalie Görtz, Susanne Axelsson, Vanessa Nolasco Ferreira, Simone Gonçalves de Assis

**Affiliations:** 1 Fundação Oswaldo Cruz, Rio de Janeiro, Brasil.; 2 vivo international e.V., Konstanz, Germany.; 3 University of Konstanz, Konstanz, Germany.; 4 University of Saarland, Saarbrücken, Germany.; 5 Kansai University of International Studies, Kobe, Japan.; 6 Instituto Nacional de Psiquiatría Ramón de la Fuente Muñiz, Ciudad de México, México.; 7 Kristiania University of Applied Sciences, Oslo, Norway.

**Keywords:** Transtorno de Estresse Pós-Traumático, Saúde Mental, Violência, Trauma, Saúde Global

## Abstract

O transtorno de estresse pós-traumático (TEPT) configura-se como um importante desafio para a saúde mental global, especialmente em contextos marcados por violência cumulativa, migração forçada e desigualdades estruturais. A Terapia de Exposição Narrativa (NET, acrônimo em inglês) é uma intervenção breve e focada no trauma, que promove a reconstrução da memória autobiográfica por meio da narrativa cronológica dos eventos de vida. Ao possibilitar a integração de experiências traumáticas fragmentadas em uma narrativa coerente, a NET facilita o processamento emocional e restaura a continuidade das histórias de vida interrompidas. Esta série de casos regionais examina a integração da NET nos sistemas de saúde mental do Brasil, República Democrática do Congo, Alemanha, Suíça, Japão, México, Reino Unido e Escandinávia. Com base em experiências diversas de implementação, o estudo identifica tanto condições favoráveis quanto desafios persistentes. Os resultados mostram que a NET é uma intervenção viável e adaptável a diferentes contextos socioculturais e níveis de recursos, especialmente quando apoiada por supervisão contínua, estratégias de deslocamento de tarefas e colaboração intersetorial. A incorporação da NET nas estruturas de serviços existentes ampliou o acesso a cuidados com enfoque no trauma baseados em evidências para populações frequentemente excluídas do tratamento especializado. Esses achados ressaltam o papel crítico de políticas públicas informadas pelo trauma na resposta às consequências da violência para a saúde mental em escala global.

## Introdução

A violência exerce um impacto profundo na saúde global, contribuindo para o desenvolvimento de transtornos relacionados ao trauma, em especial o transtorno de estresse pós-traumático (TEPT). Estima-se que 70% da população mundial vivencie pelo menos um evento potencialmente traumático ao longo da vida, e que cerca de 5,6% desenvolvam TEPT posteriormente ^1^. O peso global dos problemas de saúde mental decorrentes da violência configura um desafio expressivo, sobretudo em populações afetadas por conflitos armados, deslocamentos forçados e violência comunitária crônica; contextos em que a exposição ao trauma não ocorre como um evento isolado, mas se configura como uma condição de vida contínua e acumulativa ^2^.

O TEPT pode ter efeitos profundos e duradouros nos indivíduos, comprometendo de forma significativa seu desempenho geral e bem-estar. Os indivíduos relatam memórias recorrentes e intrusivas, entorpecimento emocional, hipervigilância e excitação fisiológica, frequentemente resultando em comprometimento funcional e isolamento social ^3^. Depressão e ansiedade são comorbidades comuns em pessoas com TEPT, trazendo maior complexidade para o quadro de saúde ^4^. Esses sintomas podem impactar severamente a capacidade de uma pessoa de atuar no trabalho, no ambiente familiar e nas relações sociais. Globalmente, o TEPT figura entre as principais causas de anos de vida ajustados por incapacidade (DALYs - *disability-adjusted life years*) ^5^. Estudos também demonstram a associação entre eventos traumáticos e uma série de condições de saúde física, como dor crônica, doenças cardiovasculares e disfunções imunológicas ^6^. Em muitos países, sobretudo os de baixa e média renda, sobreviventes de trauma continuam sem acesso ao tratamento devido ao estigma a ele associado, bem como à escassez de profissionais capacitados e às barreiras estruturais nos serviços de saúde. Essa lacuna de tratamento representa uma prioridade crítica para a saúde pública global.

Embora existam intervenções psicoterapêuticas baseadas em evidências, como a terapia cognitivo-comportamental focada no trauma ^7^, a Terapia de Exposição Narrativa (NET, acrônimo em inglês) ^8^ ^,^ ^9^ e a dessensibilização e reprocessamento por movimentos oculares (EMDR, acrônimo em inglês) ^10^, a NET se destaca por sua abordagem singular, que integra narrativa e processamento de memórias para tratar o trauma de maneira eficaz. Essa abordagem tem se mostrado particularmente útil para indivíduos que vivenciaram eventos traumáticos múltiplos ou complexos, incluindo abusos na infância e violações de direitos humanos. A NET é um tratamento breve, fundamentado em evidências e culturalmente sensível. Foi concebida para ser adaptada e aplicada inclusive por profissionais sem formação especializada em saúde mental, o que a torna especialmente adequada a contextos humanitários e regiões com poucos recursos. Estruturada em manual, a NET geralmente consiste em 6 a 12 sessões. Entre seus benefícios terapêuticos estão adaptações para crianças e adolescentes (KIDNET) ^11^, para ofensores traumatizados (FORNET) ^12^ e para comunidades (NETfatos) ^13^. Ensaios clínicos demonstraram reduções de longo prazo nos sintomas de TEPT e depressão, inclusive em cenários de violência crônica ^14^.

A NET parte do entendimento de que sobreviventes de trauma frequentemente vivenciam múltiplos eventos adversos ao longo da vida (“efeito cumulativo do trauma”), apresentando memórias traumáticas desorganizadas e fragmentadas, alterações na personalidade, autoaceitação distorcida e dificuldades de mentalização. A NET auxilia os indivíduos na construção de uma narrativa cronológica de vida, com foco nas experiências traumáticas, mas também incorporando eventos positivos significativos. Com o apoio do terapeuta com uma escuta ativa, os pacientes conseguem atribuir sentido às suas vivências, contextualizando memórias sensoriais, cognitivas, emocionais e fisiológicas/corporais, e abordando as estruturas patológicas de memória associadas aos sintomas. Ao favorecer uma reflexão sobre toda a trajetória de vida, a NET promove o fortalecimento da identidade pessoal e da autonomia ^8^ ^,^ ^9^.

Este estudo busca analisar como a NET vem sendo integrada a diferentes sistemas de saúde ao redor do mundo, com base nas experiências práticas de terapeutas e coordenadores de programas. Em vez de apresentar estudos de caso isolados, adotamos uma abordagem comparativa que permite identificar tanto padrões convergentes quanto adaptações específicas aos contextos locais. Essa decisão metodológica está alinhada às práticas recomendadas no campo da Saúde Mental Global ^15^ ^,^ ^16^. Tal abordagem possibilita revelar padrões comuns e diferenças contextuais entre países de diversas culturas, oferecendo percepção crítica sobre como o cuidado em trauma pode ser expandido de forma equitativa em sistemas com diferentes níveis de suporte institucional, disponibilidade de recursos e reconhecimento social do trauma como questão de saúde pública.

## Método

Este artigo baseia-se na prática dos autores em diferentes países ^17^. As experiências foram extraídas dos sistemas de saúde do Brasil, República Democrática do Congo, Alemanha, Suíça, Japão, México, Reino Unido e Escandinávia, selecionados por meio de amostragem em bola de neve (Quadro 1). O coordenador do projeto ou terapeuta NET de cada país respondeu a um roteiro com perguntas abertas, que contemplavam uma breve caracterização do sistema de saúde local, a formação dos terapeutas, as características dos pacientes em relação às experiências traumáticas e os desafios enfrentados durante a implementação da NET. Todos os representantes que responderam ao roteiro são autores deste manuscrito e contribuíram com a análise crítica. O objetivo foi aprofundar teoricamente situações emergentes das experiências profissionais dos autores, não havendo necessidade de avaliação por comitê de ética em pesquisa, uma vez que não envolve coleta de dados primários nem a divulgação de informações confidenciais. Os respondentes incluíram tanto profissionais de saúde inseridos formalmente nos sistemas nacionais quanto praticantes atuando em nível comunitário. Essa diversidade reflete o potencial de implementação flexível da NET, favorecendo tanto a transferência de tarefas (*task-shifting*) quanto a integração aos serviços já estabelecidos, conforme reconhecido nos marcos globais de saúde ^18^. O termo transferência de tarefas (*task-shifting*) é utilizado pela Organização Mundial da Saúde (OMS) ^18^ para designar uma estratégia de reorganização do trabalho em saúde que consiste na redistribuição de tarefas entre diferentes categorias profissionais, com o objetivo de ampliar o acesso e a cobertura dos serviços em contextos de escassez de recursos humanos. Para sua efetividade, a transferência de tarefas requer investimentos em formação, supervisão e suporte institucional, além de políticas públicas que assegurem a qualidade e a integralidade do cuidado. Em consonância com os princípios da pesquisa em saúde mental global ^16^ ^,^ ^19^, optou-se por não apresentar estudos de caso isolados, mas adotar uma perspectiva comparativa que possibilita a identificação de padrões convergentes e divergências contextuais. Essa decisão metodológica está alinhada às recomendações de síntese, em vez de fragmentação, em contextos transnacionais nos quais são necessárias intervenções em saúde mental em escala ^18^.

**Quadro 1:** Resumo da implementação da Terapia de Exposição Narrativa (NET, acrônimo em inglês) nos países analisados.

PAÍS	MODELO DE IMPLEMENTAÇÃO	TERAPEUTAS NET	RELAÇÃO COM O SISTEMA DE SAÚDE	PÚBLICO-ALVO	PRINCIPAIS PERCEPÇÕES SOBRE A IMPLEMENTAÇÃO
Brasil	Formal nos serviços públicos de atenção primária e vinculados ao sistema de justiça	Psicólogos, médicos de família e assistentes sociais	Profissionais de saúde pública do SUS	Sobreviventes de abuso sexual, violência doméstica e violência urbana	Necessidade de serviços especializados em trauma nos serviços de saúde. Articulação entre a saúde pública e o sistema de justiça como porta de entrada para uma linha de cuidado a sobreviventes de violência
República Democrática do Congo	Base comunitária via ONGs	Conselheiros comunitários	Fora do sistema de saúde formal	Sobreviventes de violência de gênero e ex-combatentes	Modelo comunitário possibilitou escalabilidade
México	Formal em instituição psiquiátrica nacional	Psicólogos e psiquiatras	Instituição pública nacional	Sobreviventes de abuso sexual e violência interpessoal	Integração sem necessidade de adaptação do protocolo
Alemanha	Formal em serviços ambulatoriais públicos/privados	Psicólogos, psiquiatras, assistentes sociais e outros	Profissionais públicos e privados	Refugiados, solicitantes de asilo e sobreviventes de violência doméstica	Cobertura por seguros facilita ampla implementação
Suíça	Integração clínica especializada em psiquiatria forense	Psicólogos e profissionais forenses	Profissionais públicos especializados em forense	Clientes forenses com transtornos mentais graves	Adaptação forense promove resiliência
Reino Unido	Modelo híbrido entre NHS e ONGs	Psicólogos, psiquiatras, enfermeiros, assistentes sociais, terapeutas	Profissionais do NHS e de ONGs	Sobreviventes de abuso, guerra e tráfico humano	Capacitação ampliou o alcance da equipe IAPT
Japão	Prática clínica privada e institucional	Psicólogos e psiquiatras	Setor privado e acadêmico	Sobreviventes de abuso, acidentes e desastres naturais	Evidências preliminares de redução de sintomas
Noruega	Serviços especializados e iniciativas comunitárias financiadas pelo setor público	Psicólogos, enfermeiros psiquiátricos e assistentes sociais	Serviços nacionais de saúde	Refugiados e indivíduos com transtornos por uso de substâncias	Atendimento culturalmente adaptado e integração com serviços cuidado em álcool e outras drogas
Suécia	Parcerias municipais locais e organizações comunitárias	Psicólogos e terapeutas capacitados em NET	Setor público e iniciativas locais	Sobreviventes de guerra, migração, violência interpessoal e organizada	Cuidado comunitário com engajamento público (p.ex.: teatro, redução de estigma)

IAPT: *Improving Access to Psychological Therapies*; NHS: sistema de saúde do Reino Unido; ONGs: organizações não governamentais; SUS: Sistema Único de Saúde.

Fonte: elaboração própria.

Nota: esta quadro sintetiza as principais características da implementação da NET em diversos contextos, com foco em modelos de integração, terapeutas e perfis de clientes para apoiar a análise comparativa entre diferentes ambientes.

## Resultados 

### Experiências de implementação da NET em diversos países

#### Brasil

O sistema de saúde brasileiro é composto por segmentos público e privado. O Sistema Único de Saúde (SUS), de caráter público, oferece serviços de saúde universais, abrangentes e gratuitos, com foco na equidade e na descentralização. Os serviços de atenção primária constituem uma porta de entrada fundamental para indivíduos com TEPT, que frequentemente se apresentam com sintomas como insônia, ataques de pânico, hipertensão e depressão ^20^. No entanto, o subdiagnóstico do TEPT na atenção primária compromete a efetividade do cuidado e contribui para o aumento dos custos em saúde ^21^. Esse cenário evidencia a necessidade de integrar, de forma mais consistente, abordagens informadas pelo trauma no sistema público de saúde.

Desde 2018, uma equipe multidisciplinar vem avançando na implementação da NET no Brasil, por meio de estudos de viabilidade e ensaios clínicos conduzidos em serviços públicos ^22^ ^,^ ^23^ ^,^ ^24^. Médicos de família, enfermeiros, psiquiatras e psicólogos receberam uma formação intensiva de duas semanas para aplicar a NET, seguida de supervisões regulares. Os pacientes traziam histórias de traumas intensos e recorrentes, como violência urbana, abuso infantil, violência sexual e violência doméstica. Muitos, inclusive, continuavam a enfrentar ameaças, como a violência na comunidade, durante ou após o tratamento. Os resultados mostraram redução clínica significativa nos sintomas de TEPT em todos os contextos avaliados, mesmo em situações de violência contínua ^24^. Em uma abordagem coletiva baseada na NETfatos ^13^, foram produzidos uma música e um videoclipe com base em uma narrativa composta pelos participantes, com o objetivo de reduzir o estigma e mudar atitudes com relação à violência doméstica (https://www.youtube.com/watch?v=iaUm7tcAoEA).

A versão adaptada da NET para ofensores, conhecida como FORNET, tem sido implementada nos Centros Judiciários de Solução de Conflitos ^25^ e em penitenciárias. A implementação da FORNET no contexto penitenciário está em curso e apresenta resultados promissores. A NET teve boa aceitação por parte dos profissionais em sua integração ao cuidado padrão. Contudo, o tempo de duração das sessões foi um desafio nos serviços de atenção primária de alta rotatividade. O caso brasileiro ilustra tanto o potencial quanto os limites da incorporação da NET em um sistema de saúde formalmente estruturado, porém sobrecarregado. As lições aprendidas ressaltam a necessidade de intervenções no nível das políticas públicas que apoiem a transferência de tarefas (*task-shifting*), o reconhecimento do trauma como problema de saúde e o financiamento de serviços especializados. Esses elementos são fundamentais para viabilizar um cuidado voltado ao trauma que seja sustentável e escalável em contextos marcados pela violência estrutural e pela desigualdade social.

#### República Democrática do Congo

A República Democrática do Congo tem enfrentado décadas de conflitos armados e deslocamentos em massa, resultando em uma carga significativa de transtornos relacionados ao trauma na população. O sistema de saúde congolês, sob gestão do Ministério da Saúde, é composto por divisões provinciais de saúde e zonas de saúde locais, que atuam como unidades primárias de implementação. As tentativas iniciais de integrar a NET ao sistema de atenção primária - por meio da formação de enfermeiros em províncias afetadas por conflitos - foram frustradas pela escassez crônica de profissionais de saúde e pela sobrecarga operacional. Em resposta, o modelo foi adaptado para uma implementação comunitária com o apoio de organizações não governamentais (ONGs) locais e organizações comunitárias. Essa mudança ilustra uma adaptação da NET que priorizou o protagonismo local, a construção de confiança e a sustentabilidade. Conselheiros comunitários, muitos deles sobreviventes de trauma, receberam formação no modelo de multiplicadores para garantir a continuidade e a escalabilidade dos serviços. Antes das formações específicas da NET, as organizações comunitárias haviam recebido treinamentos básicos em aconselhamento para apoio emocional, violência baseada em gênero e protocolos de encaminhamento, o que permitiu aos conselheiros comunitários oferecer a intervenção e registrar os casos no sistema de monitoramento das zonas de saúde.

Os projetos tiveram como público-alvo principal indivíduos com 16 anos ou mais. A maioria dos participantes havia vivenciado violência baseada em gênero e outros eventos traumáticos durante prolongados conflitos armados, e muitos também haviam perpetrado atos de violência. Além da intervenção individual com a NET, investigou-se a eficácia da adaptação comunitária da NET, conhecida como NETfatos ^13^ ^,^ ^26^. A aplicação da NETfatos em nível comunitário, aliada a uma supervisão em níveis hierárquicos (conselheiros, mestres conselheiros e supervisores), representa uma inovação robusta em transferência de tarefas (*task-shifting*) e cuidado descentralizado em trauma. Essa estrutura não apenas facilitou o restabelecimento psicossocial, mas também promoveu a ressignificação coletiva e a reconciliação. Os treinamentos variaram de 3 a 7 semanas, incluindo uma formação de três semanas em NET/FORNET com um curso de atualização de duas semanas, uma formação de duas semanas para mestres conselheiros com uma semana adicional de capacitação e uma formação de três semanas em NETfatos. Essa abordagem comunitária fortaleceu o apoio ao sistema de saúde ao reforçar o cuidado em saúde mental e a conscientização em nível local. No entanto, a instabilidade política recorrente, a violência e o deslocamento de profissionais treinados prejudicaram a continuidade dos serviços. Apesar dessas barreiras, a experiência na República Democrática do Congo demonstra como a NET pode ser mobilizada por meio de redes comunitárias.

#### México

O Instituto Nacional de Psiquiatria “Ramón de la Fuente Muñiz”, no México, é um centro de referência psiquiátrica nacional dentro da Rede Nacional de Institutos de Saúde e oferece a NET a pacientes de todo o país, com maior concentração na Cidade do México e região. Como instituição pública, o Instituto fornece o tratamento gratuitamente ou com tarifa subsidiada, conforme a condição socioeconômica dos pacientes. A integração da NET ocorreu sem necessidade de adaptações no protocolo, demonstrando sua compatibilidade com serviços estruturados de saúde mental. Os terapeutas da NET possuem formações teóricas diversas, incluindo terapia cognitivo-comportamental, terapia familiar sistêmica, terapia de aceitação e compromisso e abordagens focadas em transferência. Os pacientes atendidos apresentam uma variedade de comorbidades psiquiátricas além do TEPT e traumas complexos, como transtorno depressivo maior, transtornos de ansiedade, uso de substâncias, transtornos de personalidade e sofrimento perinatal. Frequentemente, esses indivíduos vivenciaram múltiplas formas de trauma, incluindo abuso sexual, violência interpessoal e de parceiros, violência obstétrica e violência urbana. O programa de formação em NET tem duração média de quatro a cinco dias e foi inicialmente ministrado pelo Instituto NET da Universidade de Konstanz (Alemanha).

Condições institucionais, como a infraestrutura para formação, a supervisão clínica e a colaboração interdisciplinar, contribuíram para uma oferta consistente e de alta fidelidade da NET. Os pacientes apresentaram melhorias nos domínios emocional, cognitivo e somático. Destaca-se que o processo terapêutico incorpora uma abordagem sensível ao gênero, refletindo uma atenção crescente às dimensões interseccionais do trauma ^27^. Esse modelo evidencia tanto a viabilidade clínica quanto a eficácia terapêutica da NET em contextos de cuidado especializado e, ao mesmo tempo, ressalta um desafio mais amplo na saúde mental global: garantir equidade e escalabilidade para além dos centros urbanos especializados.

#### Alemanha e Suíça

A Alemanha possui um sistema de saúde consolidado, caracterizado pela obrigatoriedade do seguro de saúde desde 2009. O sistema é estruturado em dois níveis principais: o seguro de saúde estatutário (SHI, acrônimo em inglês) e o seguro de saúde privado (PHI, acrônimo em inglês). Cerca de 86% da população está vinculada ao SHI, financiado por contribuições proporcionais à renda e compartilhadas entre empregadores e empregados ^28^, enquanto aproximadamente 10% a 11% está coberta pelo PHI. Esse sistema oferece cobertura abrangente, incluindo serviços hospitalares, ambulatoriais, de saúde mental e medicamentos prescritos ^29^. Apesar de suas fortalezas, o sistema de saúde alemão enfrenta desafios, como a fragmentação entre o cuidado ambulatorial e hospitalar, o que pode dificultar abordagens de tratamento integradas ^28^. 

O sistema de saúde suíço é semelhante ao da Alemanha, porém apresenta um grau maior de liberdade de escolha em relação a serviços específicos e acesso direto a todos os níveis de cuidado. Em ambos os países, médicos generalistas desempenham um papel crucial no apoio e no aconselhamento para doenças psicossomáticas, bem como no cuidado a indivíduos com problemas de saúde mental ^30^. No entanto, eles geralmente não oferecem psicoterapia regular ou outras intervenções psicológicas especializadas. O acesso a esses serviços especializados é frequentemente limitado, e muitos pacientes enfrentam dificuldades para obter cuidados adequados e em tempo oportuno. 

A NET é uma das terapias focadas no trauma aceitas na Alemanha, especialmente eficaz no tratamento de transtornos do espectro do trauma. Sua abordagem estruturada e centrada na narrativa tem ganhado destaque ^31^ no manejo de múltiplos traumas. A NET está incluída na cobertura oferecida por companhias de seguro e pelos serviços públicos de saúde. Diante da significativa população de refugiados na Alemanha, a NET vem sendo cada vez mais utilizada para apoiar pessoas afetadas pelo trauma. Muitos profissionais que atuam em centros de refugiados e clínicas especializadas utilizam a NET devido a sua adaptabilidade a esses contextos. Entretanto, ao longo das últimas décadas, a NET também vem ganhando importância como oferta terapêutica para a população local ^32^ ^,^ ^33^ ^,^ ^34^ ^,^ ^35^. Universidades e instituições de pesquisa alemãs, como a Universidade de Konstanz, contribuíram para a produção de pesquisas sobre a NET. Essas evidências apoiam sua eficácia, o que contribuiu para que a NET fosse aceita pelos serviços de saúde.

A integração da NET aos sistemas de saúde da Alemanha e da Suíça envolveu diversas etapas fundamentais. Inicialmente, atores estratégicos, incluindo profissionais de saúde mental, ONGs e formuladores de políticas públicas em saúde, foram mobilizados para avaliar a necessidade de intervenções focadas no trauma. Foram estruturados programas de formação com o objetivo de qualificar terapeutas em NET para a implementação eficaz da intervenção. A colaboração com diferentes instituições de saúde facilitou a incorporação da NET nos fluxos de tratamento para indivíduos em situação de trauma, especialmente no contexto de refugiados e sobreviventes de violência. Os terapeutas em NET apresentam formações variadas, sendo predominantemente psicólogos clínicos e psiquiatras, mas também assistentes sociais e outros profissionais que oferecem NET, KIDNET e FORNET em consultórios particulares, ambulatórios, unidades de internação e instituições especializadas. A duração do programa de formação para terapeutas em NET varia de dois dias a algumas semanas, abrangendo componentes teóricos e práticos para garantir que os profissionais estejam adequadamente preparados para implementar a terapia. O perfil dos indivíduos que recebem a NET inclui refugiados e solicitantes de asilo, bem como sobreviventes de abuso sexual, violência doméstica, desastres naturais, acidentes e outras experiências traumáticas.

Todos os pacientes atendidos vivenciaram traumas ou violências significativas, incluindo traumas na infância, violência doméstica, assédio moral, violência entre pares, experiências relacionadas à guerra, deslocamento forçado e violência interpessoal ^36^ ^,^ ^37^ ^,^ ^38^. Um ensaio clínico randomizado multicêntrico recente ^39^ teve como objetivo avaliar a viabilidade de integrar a NET à atenção primária, com foco no papel das equipes de médicos generalistas em sua implementação prática. O estudo apresentou resultados promissores, com boa aceitação da NET integrada ao cuidado padrão. Um projeto singular na Clínica Forense de Münsterlingen, na Suíça, utiliza a NET com pacientes traumatizados que apresentam transtornos mentais graves, como esquizofrenia e transtorno afetivo bipolar, e que foram condenados (ou absolvidos) por crimes interpessoais. O projeto reconhece que a exposição cumulativa à violência compromete a capacidade de ajustamento saudável e leva ao desenvolvimento de sintomas psicológicos.

Na Alemanha, a oferta de cuidado em saúde mental centrado no trauma e na memória traumática ^40^, utilizando a NET, está em expansão, por meio da integração de profissionais não especialistas, formados especificamente para atuar na intervenção conhecida como trabalho com as narrativas do trauma com pessoas que não apresentam nível clínico de estresse pós-traumático (NAT, acrônimo em inglês) ^41^. Essa abordagem permite que esses profissionais atuem ao lado de psicoterapeutas em um modelo de cuidado escalonado, apoiando o trabalho biográfico, a identificação precoce e as intervenções básicas para indivíduos potencialmente em necessidade. A experiência na Alemanha e na Suíça revela a introdução da NET em sistemas altamente estruturados, facilitando sua incorporação tanto em serviços especializados quanto na prática privada. No início foi desenvolvida para refugiados e populações afetadas por conflitos e atualmente a NET é utilizada de forma rotineira com indivíduos da população geral que sofrem as consequências do trauma. A disponibilidade de cobertura pelos seguros públicos e privados na Alemanha tem sustentado a institucionalização mais ampla da NET, enquanto a Suíça demonstrou como o modelo pode ser aplicado de forma eficaz no contexto da psiquiatria forense. Esses casos evidenciam que o alinhamento de políticas, a integração aos sistemas de seguro e a infraestrutura de formação são elementos essenciais para ampliar o acesso a terapias para trauma baseadas em evidências.

#### Reino Unido

O sistema de saúde do Reino Unido (NHS, acrônimo em inglês) permite que residentes, incluindo solicitantes de asilo e refugiados, acessem o cuidado em saúde mental por meio de um modelo de cuidado escalonado. O ponto de entrada para os serviços de saúde ocorre na atenção primária e nos serviços do programa *Improving Access to Psychological Therapies* (IAPT; Melhorando o Acesso às Terapias Psicológicas). Casos mais graves são encaminhados para serviços de atenção secundária ou terciária. Os serviços especializados em trauma oferecem tratamentos baseados em evidências para TEPT e TEPT complexo em todo o Reino Unido. A maioria desses serviços foi treinada e recebeu suporte para oferecer a NET, que se tornou uma opção de tratamento rotineiramente disponibilizada nesses contextos. Os serviços forenses no Reino Unido foram treinados para oferecer a FORNET, e os serviços de saúde mental infantojuvenil receberam formação para aplicar a KIDNET.

Além dos serviços do NHS, algumas ONGs também oferecem tratamentos para trauma baseados em evidências, com foco especial na formação de profissionais que trabalham com solicitantes de asilo, refugiados e sobreviventes de tráfico humano para aplicar a NET. Nos primeiros anos de formação no Reino Unido, os serviços terciários especializados em trauma foram o principal alvo para o treinamento em NET. Mais recentemente, passou-se a priorizar a formação de equipes do programa IAPT como parte de um pacote de formação mais amplo, que inclui a chamada formação complementar para os serviços do IAPT. Essa estratégia busca garantir que os terapeutas se sintam preparados para atender solicitantes de asilo e refugiados, em colaboração com organizações governamentais e não governamentais. Além de garantir maior alcance e a oferta de tratamentos para trauma baseados em evidências no nível da atenção primária, assegurando que solicitantes de asilo e refugiados tenham acesso a esses serviços para tratamento.

Os terapeutas NET incluem psicólogos clínicos, psicólogos forenses, psiquiatras, enfermeiros especializados em saúde mental, arteterapeutas, musicoterapeutas e terapeutas ocupacionais, além de assistentes sociais, psicoterapeutas e conselheiros, atuando tanto nos serviços do NHS quanto em ONGs no Reino Unido. Os programas de formação tiveram duração variável, entre dois e cinco dias, ajustando-se às necessidades específicas de cada serviço e ao nível de experiência dos profissionais envolvidos.

Adicionalmente, a supervisão clínica subsequente é oferecida de forma sistemática, assegurando a manutenção da qualidade e da fidelidade ao método. Os atendimentos abrangem diferentes perfis de pacientes, incluindo indivíduos que vivenciaram traumas múltiplos e de desenvolvimento, veteranos de guerra, solicitantes de asilo, refugiados e sobreviventes de tráfico humano, que sofreram tais experiências traumáticas em seus países de origem, ao longo do processo migratório e após a chegada ao Reino Unido.

Crianças foram atendidas com o KIDNET, enquanto clientes dos serviços forenses receberam a FORNET. Veteranos britânicos também foram tratados com a FORNET ou com a NET. Os tipos de trauma e violência experienciados pelos pacientes atendidos incluem abuso sexual na infância, negligência infantil, violência doméstica, violência comunitária, tráfico humano, traumas relacionados à guerra, agressão sexual e tortura.

Em todos os serviços, um desafio central persiste: o número limitado de terapeutas formados para atender à demanda por intervenções focadas no trauma. Apesar disso, a integração da NET nos serviços do NHS e de organizações da sociedade civil tem sido bem-sucedida ao garantir que sobreviventes de trauma recebam um cuidado eficaz e baseado em evidências. A experiência do Reino Unido evidencia a importância da colaboração entre os serviços públicos e as ONGs, assim como a necessidade de apoio político para incorporar terapias focadas no trauma aos serviços de saúde convencionais.

#### Japão

No Japão, existe um sistema universal de seguro de saúde que garante a todos os cidadãos acesso aos cuidados médicos sem barreiras financeiras. Esse modelo de seguro abrange uma ampla gama de serviços. No entanto, é importante observar que a psicoterapia permanece apenas parcialmente coberta. Dentro do atual sistema de reembolso para médicos em prática privada, a psicoterapia é limitada a, no máximo, duas sessões por mês. Essa limitação compromete o tratamento eficaz do trauma, que geralmente requer intervenções psicológicas semanais. 

Atualmente, uma iniciativa de pesquisa está em andamento para explorar a implementação da terapia de exposição narrativa no sistema de saúde japonês. Os terapeutas NET no Japão são profissionais de saúde mental, como psicólogos, psiquiatras, assistentes sociais e orientadores escolares. Eles já atuam no cuidado ao trauma em sua prática cotidiana e possuem habilidades básicas de aconselhamento, razão pela qual o período de formação em NET é de três dias, seguindo os padrões do Instituto NET (https://www.net-institute.org/). Após essa formação, aplicam a NET enquanto recebem supervisão por um determinado período. Esses esforços de formação buscam abordar a significativa escassez de cuidado com enfoque no trauma no Japão e fortalecer a capacidade da força de trabalho em saúde mental para apoiar indivíduos afetados por diversas experiências traumáticas.

A NET é utilizada principalmente para tratar sobreviventes de diversas experiências traumáticas, incluindo abuso infantil, violência doméstica, agressões sexuais, acidentes e desastres naturais. Dados preliminares da iniciativa de pesquisa em andamento sugerem que a integração da NET no Japão tem apresentado resultados promissores. Pacientes que passaram pela NET relatam uma redução significativa dos sintomas de TEPT, com muitos experimentando uma melhora clinicamente relevante. As evidências preliminares indicam melhorias clínicas nos sintomas de TEPT entre indivíduos afetados por violência sexual, abuso infantil e desastres naturais. No entanto, persistem barreiras estruturais, como a duração e a frequência das sessões de NET que, muitas vezes, são incompatíveis com as políticas de reembolso, o que dificulta a oferta sustentada fora de instituições acadêmicas ou de pesquisa. A experiência japonesa reflete uma desconexão mais ampla entre a inovação no cuidado ao trauma e a prontidão sistêmica para acomodar psicoterapias intensivas e baseadas em evidências nos sistemas nacionais de saúde. Reformas políticas, realocação de recursos financeiros e maior reconhecimento dos transtornos relacionados ao trauma são necessários para transformar o sucesso dos projetos-piloto em prática clínica generalizada.

#### Escandinávia

O sistema de saúde mental norueguês é financiado publicamente e estruturado para garantir cobertura universal de saúde. A NET foi integrada a serviços especializados e a iniciativas comunitárias ^42^. Sua implementação ocorreu por meio de colaborações nacionais que capacitaram profissionais culturalmente sensíveis para apoiar refugiados ucranianos em sua língua nativa, aumentando a acessibilidade e reduzindo a pressão sobre os serviços especializados.

A NET também foi incorporada a serviços estruturados de internação para indivíduos em recuperação de uso severo de substâncias, demonstrando sua adaptabilidade dentro de modelos de tratamento multidisciplinares. A formação e a supervisão foram coordenadas por instituições nacionais, em alinhamento com às estratégias nacionais de saúde mental, destacando o papel do cuidado com enfoque no trauma para lidar tanto com o deslocamento forçado quanto com os transtornos por uso de substâncias comórbidos.

Na Suécia, a NET foi desenvolvida por meio de parcerias municipais locais e organizações comunitárias. A iniciativa combinou a aplicação individual e em grupo da NET com ações de educação pública para reduzir o estigma e aumentar a conscientização sobre o trauma. Uma parte central da intervenção foi a produção teatral comunitária *Barrikad*, baseada nas narrativas dos participantes. O roteiro foi desenvolvido em estreita colaboração com os usuários dos serviços, garantindo que suas vozes e percepções moldassem a performance.

Terapeutas treinados em NET também formaram outros profissionais, expandindo a capacidade de atendimento. Os participantes incluíam indivíduos expostos à guerra, migração, violência interpessoal e crime organizado. Os resultados relatados incluíram redução dos sintomas de TEPT, melhora da autoimagem e maior apoio entre pares. Apesar dos resultados promissores, garantir a continuidade dos serviços com enfoque no trauma continua sendo um desafio. 

## Discussão

Este estudo sintetiza as experiências de integração da terapia de exposição narrativa em diferentes sistemas de saúde de nove países, destacando tanto a viabilidade quanto os desafios contextuais da implementação de intervenções focadas no trauma. Os resultados ressaltam a adaptabilidade da NET, abrangendo desde modelos centralizados e institucionais até abordagens descentralizadas e comunitárias, evidenciando sua capacidade de reduzir sintomas em contextos marcados por violência, escassez de recursos ou fragmentação sistêmica.

O Quadro 1 apresenta uma visão comparativa dos modelos de implementação, perfis dos terapeutas e trajetórias de integração aos sistemas de saúde. Países como Alemanha, Reino Unido e México demonstram como sistemas públicos estruturados podem institucionalizar a NET em serviços especializados, enquanto República Democrática do Congo oferece exemplo relevante de transferência de tarefas (*task-shifting*) e disseminação comunitária. A variação nos modelos de implementação reflete a prontidão sistêmica, a capacidade da força de trabalho e as estruturas de governança em saúde mental já existentes. Isso reforça as recomendações da OMS ^18^ para modelos contextualmente responsivos, que conciliem rigor clínico e realidades sociopolíticas.

Apesar do sucesso clínico, um desafio recorrente foi a tensão entre a fidelidade à técnica e a escalabilidade nos sistemas de saúde. No Brasil e no Japão, por exemplo, a duração das sessões da NET entrou em conflito com o fluxo de trabalho dos serviços gerais de saúde e com as políticas nacionais de reembolso. Essa situação evidencia um desalinhamento estrutural entre os protocolos psicoterapêuticos baseados em evidências e a lógica operacional da maioria dos sistemas públicos de saúde, especialmente em contextos com recursos limitados.

A experiência do México demonstra tanto a viabilidade clínica quanto o potencial terapêutico da NET em serviços especializados, mas também evidencia a necessidade urgente de integrar abordagens focadas no trauma ao sistema de saúde mental como um todo. Para garantir o acesso equitativo, as intervenções focadas no trauma devem ser apoiadas por políticas nacionais, financiamento adequado e sistemas de supervisão clínica que alcancem áreas além dos serviços de referência nos grandes centros urbanos. A implementação descentralizada surge como uma estratégia viável para ampliar os serviços de saúde mental com enfoque no trauma, especialmente em áreas vulneráveis e pouco assistidas ^16^ ^,^ ^18^ ^,^ ^43^. A atenção primária, os serviços escolares e as ONGs constituem pontos de entrada essenciais para modelos descentralizados e escaláveis. Recomendações globais ressaltam que transformar significativamente os sistemas de saúde mental exige transferência de tarefas (*task-shifting*), articulação política e mecanismos de financiamento que sustentem o cuidado com enfoque no trauma para além dos contextos acadêmicos e urbanos ^43^.

A sensibilidade cultural e a capacidade de adaptação ao contexto foram fundamentais para a integração eficaz da NET nos sistemas de saúde. As adaptações linguísticas e culturais figuram entre as modificações mais recorrentes. A supervisão clínica pós-formação permanece essencial para garantir a fidelidade ao tratamento e a sustentabilidade a longo prazo.

Na República Democrática do Congo, a implementação inicial incluiu uma formação intensiva, seguida de uma supervisão estruturada entre pares e da criação de uma rede local de profissionais treinados. Essa estratégia reforçou a garantia de qualidade, fortaleceu a capacidade local e promoveu o protagonismo dentro do sistema de saúde.

Do ponto de vista metodológico, este estudo adotou uma perspectiva comparativa para identificar convergências e divergências entre as experiências nacionais. Essa escolha está alinhada às recomendações da literatura em saúde mental global, que propõe a síntese em vez da fragmentação nas pesquisas sobre implementação ^15^ ^,^ ^18^. A utilização de um modelo estruturado para a apresentação dos dados, conforme mostrado no Quadro 1, possibilitou a geração de *insights* entre os diferentes contextos, ao mesmo tempo em que manteve a atenção às especificidades sociopolíticas e institucionais de cada país.

As experiências escandinavas demonstram que, mesmo em sistemas estruturalmente robustos, ainda existem desafios para institucionalizar o cuidado com enfoque no trauma. Na Noruega, a implementação ocorreu por meio de esforços coordenados entre serviços especializados e iniciativas comunitárias financiadas com recursos públicos. Isso evidencia como a infraestrutura de políticas, quando combinada a sistemas de supervisão e planejamento de longo prazo, pode facilitar a integração sustentável da NET.

Na Suécia, estratégias de conscientização pública, incluindo iniciativas participativas como o teatro comunitário, foram integradas ao cuidado clínico para reduzir o estigma e fomentar o engajamento. Embora essas abordagens tenham mostrado resultados promissores na redução de sintomas e na reintegração social, a ausência de uma estratégia centralizada para o trauma limitou a escalabilidade.

Embora o estresse traumático represente uma preocupação crescente para a saúde pública global, a maior parte das pesquisas e publicações sobre o tema tem refletido, de maneira desproporcional, os contextos de países de alta renda ^44^. Esse viés geográfico na literatura acadêmica sobre trauma e TEPT limita a compreensão das experiências e das necessidades em saúde mental das populações afetadas pelo trauma em países de baixa e média renda, onde o impacto da violência, dos conflitos e de outras formas de adversidade é frequentemente mais acentuado. Enfrentar esse desequilíbrio na base de evidências é fundamental para desenvolver estratégias de prevenção e tratamento de transtornos relacionados ao trauma que sejam abrangentes, culturalmente sensíveis e pertinentes no cenário global.

Sob a perspectiva da saúde mental global, a NET representa uma intervenção culturalmente sensível para os transtornos relacionados ao trauma, especialmente em contextos com poucos recursos e em cenários pós-conflito. Seu formato breve e estruturado, aliado à capacidade de abordar múltiplos eventos traumáticos, responde às necessidades de populações expostas a guerras, violência e desastres naturais. A abordagem baseada em narrativas dialoga com práticas universais de contação de histórias, ampliando sua relevância cultural. O foco da terapia na documentação de violações de direitos humanos pode, ter implicações sociopolíticas mais amplas ^8^ ^,^ ^9^.

À medida que os esforços em saúde mental global buscam ampliar o acesso a tratamentos baseados em evidências, a adaptabilidade e a eficácia da NET a tornam uma ferramenta valiosa. Desenvolvida a partir de pesquisas ^14^, a NET deve ser acessível em todos os países, inclusive em contextos com recursos limitados. A duração e a intensidade da formação são, portanto, flexíveis, dependendo da formação terapêutica prévia e da experiência do profissional. A transferência de tarefas (*task-shifting*), mais do que uma regulamentação rígida de aplicação e disseminação, torna a NET viável em contextos de alta demanda, emergências ou escassez de recursos.

Uma limitação deste relato de experiências é seu escopo geográfico restrito, concentrando-se em um número limitado de países. Metanálises indicam que ensaios clínicos com a NET já foram conduzidos em pelo menos 30 países ^14^, o que sugere um potencial para uma implementação ainda mais ampla da terapia. Portanto, são necessárias pesquisas adicionais para aprofundar a compreensão sobre a implementação da NET em contextos nacionais diversos.

De modo geral, as evidências apoiam a NET como uma abordagem eficaz do ponto de vista clínico e com flexibilidade estrutural para o cuidado em trauma. Seu caráter não comercial, a capacidade de adaptação à transferência de tarefas (*task-shifting*) e o alinhamento com os objetivos de equidade em saúde global fazem da NET uma candidata promissora para iniciativas de ampliação em escala nacional. No entanto, o êxito na implementação depende não apenas da eficácia terapêutica, mas também de investimentos sistêmicos, formação profissional e inovações ajustadas aos contextos locais.
